# Occupational Health and Safety and Migrant Workers: Has Something Changed in the Last Few Years?

**DOI:** 10.3390/ijerph19159535

**Published:** 2022-08-03

**Authors:** Stefano Porru, Michela Baldo

**Affiliations:** 1 Department of Diagnostics and Public Health, Section of Occupational Health, University of Verona, 37134 Verona, Italy; michela.baldo@aovr.veneto.it; 2 Clinical Unit of Occupational Medicine, University Hospital of Verona, 37134 Verona, Italy; 3 MISTRAL-Interuniversity Research Centre ‘Integrated Models of Study for Health Protection and Prevention in Living and Working Environments’, University of Brescia, Milano Bicocca and Verona, 37134 Verona, Italy

Over the last few decades, the migratory phenomenon, supported by social, economic, and political aspects, has become progressively intensified and structured, increasing its relevance as a topic of interest for researchers of various disciplines and, among these, occupational health and safety exerts a prominent role.

While it is always difficult to obtain real numbers in this area because the sources of the information are often organizations with other missions, current global estimates provided in 2022 by the World Health Organization highlight that from 1990 to 2020 the total number of international migrants increased from 153 to 281 million people, and, especially in the last decade, the greatest increases were due to family and labor migration [1].

According to these numbers, information collected by Caritas and Migrantes also shows that the global migrant population constitutes 3.6% of the entire population of the planet [2], growing by about 108 million in twenty years and 9 million in a single year, despite the closing of borders to mitigate the spread of COVID-19.

It is clear that the global number is inevitably destined to also increase in the years to come, induced by the demographic aging and labor demand in the most developed countries, and further fueled by the consequences of the SARS-CoV-2 pandemic, the gradual economic recovery and reopening of the job market, and, last but not least, the recent dramatic geopolitical events, such as the current war in Ukraine.

Since the search for work opportunities is one of the main reasons for undertaking the migration path, the so-called “economic migrants” represent the vast majority of international migrants, making up about two thirds of them, with 169 million in 2019, which is equivalent to approximately 5% of the global workforce [1].

Middle–high-income European countries are one of the first destinations on migration routes, and in the last 20 years, they have shown a more sustained immigration growth, hosting about 35% of the international migrant population.

Among the EU-27 countries, there were in fact around 37 million resident foreigners present (8.3% of the total population), 5 million of whom were welcomed by the Italian state.

In Europe, these resident foreigners represent approximately 17% of the workforce.

The considerable economic potential of migrants is recognized across the board, and it is expressed both in the country of origin, which can benefit from the sort of help from outside through remittance flows, and in the country of settlement, where an important contribution to local productivity is guaranteed.

Although the phenomenon is now deep-rooted over time, there are still differences with the native population in participation in the labor market to this day.

Migrant workers are still required, and they are employed to fill the demand in those sectors and those jobs least favored by the native-born, for which, consequently, there is a shortage of local labor.

Most of these are the so-called 3D jobs (dangerous, dirty, demanding/degrading). The roles offered include precarious positions in the labor market; the absence of contracts due to irregular employment or temporary assignments; the tasks assigned being among the most burdensome, arduous, manual, physically demanding; the schedules and shifts proposed often being unfavorable, with demand for flexible work and sustained rhythms; the positions available requiring low skills, often disproportionate to the real level of experience or qualification; and the salary generally being low.

The uncertain nature of the employment relationship and the lack of bargaining power, legal defense, and information are in particular some of the aspects that bring about a disadvantage and exacerbate the difficulty in expressing requests concerning better working conditions, often for fear of retaliation, negatively affecting the possibility of exercising one’s rights [3].

There are also differences in the employment rate, which was about 14 percentage points lower in foreign citizens compared to European citizens in 2019, with an unemployment level of 14.7% for non-EU citizens.

Common situations characterized by poor safety standards, reduced availability of protective gear, and lack of information, education and safety training do not allow workers’ health to be adequately safeguarded; irregular work, in particular, also emphasizes migrants’ vulnerability, exposing them to the most serious risk of abuse and exploitation.

It is therefore undeniable that these workers are disproportionately relegated to perform hazardous activities in poor working conditions compared to the general population, and these aspects can endanger their health and safety and contribute to the onset of pathologies [4].

The European Working Condition Survey recently revealed a more frequent exposure to occupational risks among migrants compared to native workers: during their work, they are often affected by high temperatures, loud noises and vibrations, the maintenance of an upright position for a long period of time, or faster work rates.

Furthermore, migrant workers tend to be employed in activities involving increased exposure to pesticides and chemicals, especially in the agricultural sector.

In the background, aspects related to living and social conditions and behavior, such as isolation and poor support, linguistic and cultural barriers, a different perception and underestimation of potential dangers, and the tendency to take higher risks, make these groups more vulnerable and suggest considering the migrant status a real health determinant.

Moreover, migrants suffer from inadequate care and unstructured health surveillance services; they struggle to access the health system and are excluded from community and insurance protections, which are essential elements for social, economic, and political development and preconditions for achieving global health goals [3].

Epidemiological evidence has found, in fact, a greater prevalence of worse mental and general health conditions in the migrant population, arising from the trauma suffered during the migration process and sometimes after the arrival in the host country, a greater perception of discrimination and reporting of high levels of anxious-depressive disorders, and a higher incidence of psychotic disorders, resulting from adverse socio-environmental conditions and experiences [5].

The paths, mechanisms and global impact are not well known; however, it seems that employment conditions and work organization could actually be dangerous for migrants’ health.

Despite the shortage of published studies, most of the various reports show that migrant workers are exposed not only to a high occupational risk but also to frequent and severe injuries.

The International Labour Organization (ILO) estimates that there are 2.78 million deaths every year due to occupational injuries or illnesses, for a total cost appraised on average at 4% of the gross national product.

Fatal accidents at work make up about 14% of cases, while the greatest share of deaths (more than 3/4) are attributed to circulatory diseases, malignant neoplasms and respiratory diseases (31%, 26%, and 17%, respectively) [6].

Work-related diseases are hard to track and therefore generally underestimated due to their latency and potentially multifactorial genesis; in addition to the aforementioned, a fair number are also constituted by musculoskeletal disorders and psycho-social problems.

Official statistics of the National Italian Insurance Institute for Workers’ Occupational Injuries and Diseases (INAIL) recorded about 645,049 total work-related injuries in 2018: 16.3% of them were attributable to migrant workers (18.8% in the following year). There were 1218 fatal accidents, 17% of which concerned migrants. Furthermore, a progressive increase was observed in reported accidents in this group in contrast to what has been noticed in natives.

As for occupational diseases, every year, approximately 1800 cases are reported in foreign workers, showing a progressive increase as well.

In 73% of the countries whose data are reported in the ILO statistical databases, the incidence rate of fatal occupational accidents was higher in migrants than in the native population: this disproportion largely comes from the distribution of occupations among migrants, who are excessively represented in those jobs with an increased rate of injuries.

Even the international literature often reports a higher risk of total and fatal injuries in migrant workers compared to natives [7].

Some authors underline that migrants are on average involved in occupational accidents at least twice as often compared as nationals [8].

A non-systematic review of the scientific literature has highlighted a risk of incurring fatal events from 2 to 5 times higher in immigrant workers, regardless of the working sector, than in local population; a significantly high risk was then documented in immigrants employed in specific sectors such as agriculture [5].

The field of agriculture, in particular, shows the highest levels of fatal injuries in the US; global data on occupational accidents are limited but tend to confirm the results of these studies.

Non-fatal accidents also seem to be more common among working immigrants, although data are less consistent.

In agriculture, however, there is a high proportion of undeclared or informal work arrangements, which certainly enhance these issues.

Considering work-related diseases, in this context, a significant prevalence of some of them, in particular musculoskeletal disorders, skin, respiratory and heat pathologies, hearing loss from noise, and infections and poisoning from pesticides, has also been detected, more frequently in immigrant workers than natives (85% vs. 15%), related to the nature of the activity.

With the most recent political and health events, some disadvantageous conditions have also worsened, and social insecurities and inadequacies have been highlighted.

The last two years have been characterized by one of the most severe pandemics of the current century, with a systemic impact on mobility and migration.

COVID-19 has radically altered mobility around the world: the restrictions imposed for the prevention and control of the spread of the infection by various countries have limited international migration, making it no longer possible to send remittances to the family, increasing return migration and having a huge influence on the total workforce.

The pandemic has contributed to a worsening of the employment and working conditions of immigrants, which is overrepresented in essential sectors and most affected by the pandemic, increasing unemployment rates more radically than in the native population, and it has also exacerbated the already existing inequalities, as measures to mitigate the effects and assist the population do not include their group.

This situation is amplified for undocumented workers, who are excluded from social security programs, compensation, and subsidies.

Thus, migrant workers are potentially both disproportionally more affected by the impact of COVID-19, particularly in lower paid jobs, and part of the response to the pandemic, working in critical sectors [9]: undertaking high-risk occupations, they enable native workers to move into work with fewer face-to-face interactions, serving as a sort of protective shield [10].

Migrant workers are more likely to be infected—at least twice the risk of the native-born—because of several factors: physical distancing or teleworking are rarely applied to essential activities in which they are mainly involved; inadequate hygiene standards and crowding working and living conditions are common; information, knowledge, support of effective health and safety protection measures, and access to job protection are limited; and the poor conditions in origin and transit countries increase their risk [11].

In early April 2020, for instance, a marked escalation in daily numbers of new cases of COVID-19 was observed among low-skilled migrant workers living in dormitories in Singapore, highlighting the need to address inadequate housing before any pandemic occurs [12].

Moreover, many countries depend on foreign-born workers in the critical sector of healthcare services.

Van der Plaat et al. estimated a relative risk of an absence of COVID-19 sickness in National Health Service staff in England between 1.5 and 2.5 among most essential workers in patient-facing occupations, suggesting an improvement in risk reduction strategies [13].

Additionally, in Singapore’s construction sector, migrant workers were disproportionately affected, as they shared accommodation and worksites, making it necessary to introduce multilevel safe management measures to enable the reopening of the activities [14].

Furthermore, the effectiveness of diagnosis, tracking and follow-up, continuity of care, and correct quarantine and isolation measures are not always promptly or adequately pursued.

Migrants, and especially irregular workers, were shown to have been less tested, in relation with the possible consequences of a positive outcome.

A systematic analysis of the international literature confirms the greater exposure to risk in ethnic minorities and worse clinical outcomes from COVID-19 due to cultural, behavioral, and socio-economic differences [15].

Migrants employed in the agricultural sector are particularly susceptible to infection, whose transmission is made easier by frequent close contacts during work and by sharing means of transport and housing.

The impact of the pandemic and its consequences, such as the risk of job loss, loss of income, and stigmatization, have affected the physical and mental health of migrants, as several studies have proved [16].

The COVID-19 crisis also hits social integration, putting even children of migrants at a disadvantage in several ways.

In addition, migrants and refugees have suboptimal vaccination coverage compared to the general native population: they are generally excluded from health vaccination plans and lack cultural sensitivity and outreach, and misinformation, cultural beliefs, vaccination costs, and high mobility represent some of the several access barriers they can encounter. Across the different destination countries, wide heterogeneity emerges for entitlement to vaccination, which is at the core of the public health response.

Since a strict interdependence is recognized between migration and public health, rapid and universal access to available vaccinations such as a healthy equity interventions to protect both the vulnerable groups and the entire community is recommended and also affirmed by Sustainable Development Goals.

For the reasons listed above, G20 countries should include migrants in national COVID-19 response and vaccination plans, encouraging access among undocumented people, monitoring disparities and measuring impact, increasing cultural sensitivity and competence, establishing literacy education programs and strategies, offering health promotion interventions, and monitoring progress [17].

Even recent geopolitical events, such as the war in Ukraine, have contributed to causing the displacement of thousands of people and to determining further instability in migratory flows.

In light of the growing importance of the phenomenon, some countries have begun to promote cohesion policies focused on migrant populations.

The World Health Organization has prepared a comprehensive action plan, whose priority is to ensure health coverage, increase universal social protection, and reduce xenophobia and discrimination.

The 2030 Agenda for Sustainable Development adopted in 2015 by the member states of the United Nations has recognized the positive contribution made by migrant populations and has developed inclusive strategies, objectives, and actions in order to reduce poverty and leave no one behind, increase the empowerment of disadvantaged and marginalized individuals, and focus its efforts to achieve a full and productive employment and decent work for all.

The identified goals have the purpose of limiting inequalities and implementing programs for the good management of migration, mobility, and integration; recent studies suggest that countries where integration is greater have better socio-economic and health outcomes [18].

Nonetheless, concrete actions aimed at studying conditions of disparity, detecting related causes, and identifying and managing migrants’ needs, which are indispensable for improving preventive and corrective interventions, are still unsatisfactory.

There is still a shortage of efficient systems to fill cultural, linguistic, and information gaps; services designed to make up for the insufficient support due to isolation and social exclusion are still poor; health, physical, and mental problems remain scarcely investigated; there is a lack of prevention and timeliness of diagnosis; the breaks in the continuity of care are often attributable to constant relocation; and the difficulty of accessing care services remain to be evaluated.

The approach itself is actually complex, since it deals with a non-homogeneous population, generally excluded from official statistics or whose data, when considered, are not disaggregated.

At present, there are no global or national indicators related to migrant health, and no regional system has been implemented for standardized data collection, so studies often do not consider large segments of the population and incorrectly report the health status in surveillance surveys.

This leads to a paucity of consistent, scientifically valid, and comparable health data on migrants.

The category of migrant workers in particular remains a category that is still scarcely visible in the eyes of public policy, although it deserves the same guarantees as compared to non-essential workers, with significant social costs and health repercussions.

The Occupational Medicine Health and Safety System has not substantially changed: quality standards are lacking; information and health education, risk assessment, adequate health surveillance, and case management are not completely provided; and an analysis on the connections with working conditions is missing.

For these reasons, challenges to bridging the gap in the knowledge of the relationship between employment and health still remain.

A basic obstacle is first and foremost the difficulty of access among migrant populations, in particular due to the large portion of irregular work, transient employment, the use of labor intermediates, and the mobility, which hinders data collection.

There is a clear need to improve the system for reaching this vulnerable population; approaches using outreach activities seem convenient, including active and mobile interventions aimed at intercepting and create a contact not only directly inside the workplace, but also outside in the places of aggregation or marginalization. New techniques must be developed, and various sources of interception, spread throughout the territory, should be considered, such as where migrants arrive (hosting centers, humanitarian organizations, patronages, trade unions, training or employment centers, trade associations, and places of access to care).

Once migrants are reached and hooked up, it is necessary to spread information and introduce and deploy tools, such as risk assessment, health surveillance, health promotion, and, last but not least, effectiveness assessment of interventions, which are aimed at improving their working conditions.

To date, the accessibility to information is variable; most countries have not adopted adequate national monitoring systems concerning key occupational health problems, and those that do use different categorizations and definitions.

Most of the relevant studies come from the US, and only a few distinguish migrant groups by ethnicity and work background.

The European scientific literature currently has a limited number of studies and reviews, whose data focus mainly on occupational accidents: the statistics available, however, are not uniform in terms of definitions, collection methodology, and data quality.

Prospective cohort studies are missing, and many studies are cross-sectional and rely on self-reported information. Available data are not only scarce but often collected in different time intervals, based on samples, and do not provide certainty of the validity of the tools and methods used to measure occupational exposure. The lack of accuracy and consistency in assessment methods makes it difficult to compare the risk and prevalence between different studies.

It is evident that the relationship between health, work, and immigration is still understudied, and occupational injuries and work-related illnesses remain underdiagnosed, underestimated, or misclassified.

It is therefore desirable to set up inclusive policies and public health interventions that will help to smooth out inequalities, through the encouragement of investments in linguistic literacy, support and translation services, enhancements to the transmission of information and skills, raising health awareness and education, improving social services and accessing health services, and favoring a greater collaboration with migrant population management organizations and agencies in order to promote healthy practices and lifestyles.

Considering the workplace, migrant-focused risk assessment and risk management, with technical, organizational, procedural prevention measures, health surveillance, and fitness for work, are undoubtedly to be enhanced and implemented, for the purpose of both improving the quality of migrant workers’ working life and promoting their health, within the framework of corporate social responsibility.

It is necessary, first of all, to develop conceptual models to understand the causal mechanisms of empirical evidence.

Therefore, tools and procedures that include migrants in the data collection processes, such as censuses, national statistics, and surveys, are certainly useful, as well as innovative methods to capture the hidden part of the population.

In Europe, large-scale studies on cultural background and socio-economic aspects, language skills, length of time spent in the country, and reliable estimates of migratory flows would be essential.

There is also no doubt about the need to monitor the health status of the migrant population through the collection of standardized and comparable data, to map good practices, and to create a “migrant-sensitive” health system, in order to incorporate migrants’ needs and introduce ameliorative interventions.

In addition, knowledge of working and employment conditions is essential for verifying causal relationships and evaluating improvement hypotheses by means of the development of intersectoral studies and preventive programs.

For this purpose, it is of prime importance to promote harmonization within countries and establish information and surveillance systems that allow a systematic collection of comparable and high-quality data.

New tools and measures should be developed that are capable of investigating the relationship between working conditions and occupational health and the nature and causes of inequalities and are able to analyze the specific mechanisms responsible for damage to health through work.

Analytical research is fundamental to understanding the intermediate processes between employment, working conditions, and psychophysical health and behaviors, with models that specify how much the macroeconomic developments of various countries, employment situations, and health can be correlated.

Better estimates of the risks of injury, illness, mortality, and multicenter studies that integrate data, overcoming the fragmentation of current studies and reducing local differences, would be useful.

It is therefore essential to create more powerful and better epidemiological designs, integrating quantitative and qualitative data, to improve the comprehension of the pathways, mechanisms, and correlations between work and health inequalities and to evaluate the effectiveness of interventions.

Hence, it is necessary to put into practice all the solutions with concrete interventions and to put into play all the available tools, precisely in the places where the events happen, acting directly in every single company or workplace or situation, focusing attention on all types of cases, be they refugees, regular workers, or persecuted, trying to actually improve the whole prevention system.

Three years ago, with the Special Issue of *IJERPH* entitled “Migration, Work and Health”, we attempted to tackle the many complex aspects pertaining to the relationship between migration, work, and health, including individual susceptibility and vulnerability; working mood and conditions; occupational risk exposure; the importance of information, education, training, and adequate communication; health diseases; and access to care [19].

In these last few years, SARS-CoV-2 has had a significant impact on migrant workers, increasing their vulnerability and changing and reorienting all the checks and preventive measures in the workplace, and the outbreak of further unforeseen geopolitical events has contributed to changing migration flows and economic balances.

We still believe that there is a strong need to implement health and safety in every workplace, through risk assessment, risk management, health surveillance, and fitness for work focused on migrant workers, in order to improve their quality of working life and promote their health, within the framework of corporate social responsibility.

In this second edition of the Special Issue, therefore, we are focusing again on this topic, providing an updated, multidisciplinary, and evidence-based overview of the state of the art of the occupational health and safety of migrant workers worldwide, covering a comprehensive range of primary, secondary, and tertiary prevention. We would like to offer researchers and experts the opportunity to publish their original work, especially in the field of occupational health, public health, epidemiology, social sciences, health economics, and international labor law. Data from field studies, intervention effectiveness studies, and reviews related to this research area, with special attention paid to the impact of SARS-CoV-2 and the COVID-10 pandemic on migrant workers and workplaces, are particularly welcome.
